# Ascariasis, Amebiasis and Giardiasis in Mexican children: distribution and geographical, environmental and socioeconomic risk factors

**DOI:** 10.1007/s12639-020-01260-2

**Published:** 2020-08-13

**Authors:** Gerardo A. Zavala, Eline van Dulm, Colleen M. Doak, Olga P. García, Katja Polman, Maiza Campos-Ponce

**Affiliations:** 1 Department of Health Sciences, Faculty of Science, Vrije Universiteit Amsterdam, Amsterdam, The Netherlands; 2 Department of Health Sciences, University of York, York, UK; 3 Facultad de Ciencias Naturales, Universidad Autónoma de Querétaro, Santiago de Querétaro, Mexico; 4 Department of Public Health, Saint Ambrose University, Davenport, IA USA; 5 Department of Biomedical Sciences, Institute of Tropical Medicine, Antwerp, Belgium

**Keywords:** Intestinal parasites, Ascariasis, Amebiasis, Giardiasis, Mexico, Childen

## Abstract

The aim of this study is to provide an overview of the geographical distribution of Ascariasis, Amebiasis and Giardiasis, and to identify specific geographical, socioeconomic and environmental factors that are associated with the incidence of these infections in Mexican children. We made use of publicly available data that was reported by federal organizations in Mexico for the year 2010. The contribution of geographical, socioeconomic and environmental factors to the incidence of infections was assessed by a multivariable regression model using a backwards selection procedure. *A. lumbricoides* incidence was associated with mean minimum temperature of the state, the state-wide rate of households without access to piped water and toilet, explaining 77% of the incidence of *A. lumbricoides* infections. Mean minimum precipitation in the state, the rate of households without access to a toilet, piped water and sewage system best explained (73%) the incidence of *E. histolytica* infections. *G. lamblia* infections were only explained by the latitude of the state (11%). In addition to the well-known socioeconomic factors contributing to the incidence of *A. lumbricoides* and *E. histolytica* we found that temperature and precipitation were associated with higher risk of infection.

## Introduction

Intestinal parasitic infections are a public health problem in Mexico (Gutiérrez-Jiménez et al. 2017). While infection can occur at any age, school age children (5–9 years) are most at risk for intestinal parasitic infection, due to their behaviour and increased exposure (Zavala et al. 2017), and they are at the highest risk of morbidity among all age groups(Buonsenso et al. 2019). Intestinal parasites can be divided into soil transmitted helminths (STHs) and intestinal protozoa. In Mexico the most common STH is *Ascaris lumbricoides (A. lumbricoides)* with a prevalence between 16% and 33% depending on the region of the country(Gutierrez-Jimenez et al. 2013; Medina et al. 2013). Even though in many cases *A. lumbricoides* infection is asymptomatic, it has been associated with stunting, anemia, reduced physical fitness, respiratory and gastrointestinal complications (Hotez et al. 2008). For these reasons, the surveillance epidemiological system of Mexico (SINAVE) requires all *A. lumbricoides* cases to be reported.

The most prevalent intestinal protozoa in Mexico is *Entamoeba coli*. However this parasite has been categorized as a non-pathogenic protozoa and is therefore not reported in the epidemiological surveillance system of Mexico (SINAVE)(Speich et al. 2013). *Entamoeba histolytica* (*E. histolytica*) and *Giardia lamblia* (*G. lamblia*) on the other hand, are responsible for malabsorption, diarrhea, blood loss and reduced growth, and thus SINAVE requires case notification for these two pathogenic intestinal protozoa (Rossignol et al. 2001).

Both STH and protozoa infections occur by fecal–oral transmission (Shumbej et al. 2015). STH eggs require embryogenesis in the soil to become infective, and to achieve this they need specific environmental conditions, related to soil humidity, temperature, rainfall, vegetation density and type of climate (Gunawardena et al. 2004; Saathoff et al. 2005). Environmental and socioeconomic (e.g. poverty, sanitation, education)(Ziegelbauer et al. 2012) determinants have been shown to be associated with parasitic infections (Norhayati et al. 1998; Gunawardena et al. 2004; Saathoff et al. 2005; Scholte et al. 2012; Schule et al. 2014; Shumbej et al. 2015), but with important differences between countries or regions (Saathoff et al. 2005; Scholte et al. 2012; Welch et al. 2016).

To the best of our knowledge, there are no country-wide studies on the geographical distribution, and socioeconomic and environmental risk factors of intestinal parasites in Mexico. This paper therefore aims to provide an overview of the distribution of the most important parasitic infections, and to identify geographical, socioeconomic and environmental factors that are associated with the incidence of these intestinal parasitic infections in Mexican children.

## Methods

### Study design

For this ecological study we created a database containing publicly available data from the 32 states covering the whole territory in Mexico. The database included information on the state-wide incidence of different intestinal parasites in all children aged 5 to 9 years, and associated environmental and socioeconomic variables. We selected the most recent available data (i.e. 2010), in which all the relevant variables were publicly available.

#### Intestinal parasitic infection incidence

The incidence of intestinal parasitic infections (*A. lumbricoides, E. histolytic*a and *G. lamblia)* were obtained from the SINAVE from children aged 5 to 9 years. The data is publicly available at: http://www.epidemiologia.salud.gob.mx. Medical doctors of all health facilities of the country report laboratory confirmed cases of *A. lumbricoides, E. histolytica/dispar* and *G. lamblia* infections by age group through the SINAVE webpage, which is a national reporting system following standard procedures to ensure the quality of the data (Tapia-Conyer et al. 2001b). Cases are reported as the incidence per 100,000 person-year for each age group for each of the 32 states of Mexico (Tapia-Conyer et al. 2001a). All other helminth infections are reported in SINAVE as “other helminth infections” and all the other intestinal protozoa infections are reported as “other protozoa infections”(Buck 2013). The ‘other’ categories were not analyzed in this study because the “other helminths” reflect a combination of 15 helminths with different infection routes and hosts, and the “other protozoa” combine 5 different pathogenic and non-pathogenic protozoa (Bethony et al. 2006).

#### Geographical and Environmental variables

We selected all the available geographical and environmental variables that are known to be associated with intestinal parasitic infections (Saathoff et al. 2005; Scholte et al. 2012). State-wide data of the annual temperature (°C) of the state, annual precipitation (mm), latitude (°), mean altitude (m) and the percentage of warm-humid climate (%) of each of the 32 states were obtained from the National Institute of Statistics and Geography (INEGI) 2010 climate report. Temperature and precipitation were gathered and reported by the INEGI as the mean minimum, mean maximum and average annual temperatures and precipitation of the last 30 years for each state, from over 3758 available weather stations across the country. Latitude was defined as the latitude in the centroid of the state and the percentage of warm-humid climate was directly extracted of the 2010 INEGI report. Warm-humid climate was defined as a region with annual average temperature over 18 °C with precipitations all year long (https://www.inegi.org.mx/temas/climatologia/).

#### Socioeconomic variables

We selected all available socioeconomic variables that are known to be associated with parasitic infection in other studies (Kightlinger et al. 1998; Ziegelbauer et al. 2012; Strunz et al. 2014; Speich et al. 2016). For each state we extracted data on the mean age of the population, rate of the population with health coverage, the rate of households living in poverty, living in extreme poverty as well as the rate of households without access to sewage system, piped water, and toilet were collected by the National Institute of Statistics and Geography (INEGI) in the countrywide population census, which is publicly available at http://www.inegi.org.mx/ (Instituto nacional de estadística 2012).

#### Statistical analysis

Univariable models were performed to assess the specific associations of environmental and socioeconomic variables in each state with the state-wide incidence of each intestinal parasite studied. Thereafter, variables that were associated with the outcome (*p* < 0.15) were selected to be used in a multivariable linear regression model, one for each parasite. The best multivariable model was obtained with a backward procedure with an with an entry level of 0.15 and a threshold for inclusion into the final model of 0.1 (Draper et al. 1966). We assessed the model by the goodness of fit (R^2^). For internal validation we obtained bootstrapped estimates (regression coefficients, *p* value and goodness-of fit) and compared these changes to the empirical dataset (Brunelli 2014). All statistical analyses were performed using SPSS version 21 (SPSS, Chicago IL).

Intestinal parasites were mapped according to their incidence in 5 groups (quintiles). The unit of mapping was “the state” the largest administrative unit of Mexico. The maps were generated using R studio ggplot2 package (Boston, MA).

## Results

**Fig. 1:**
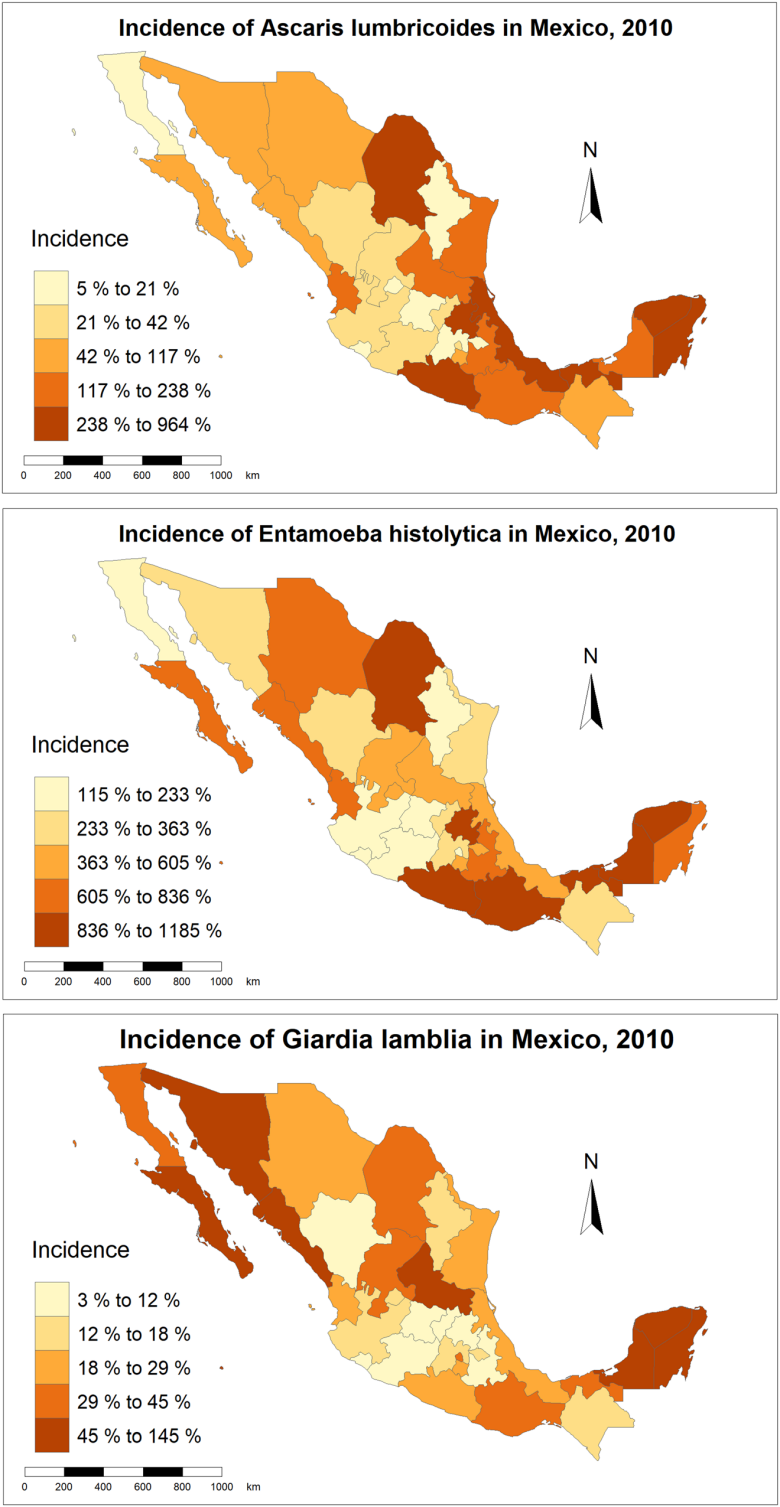
Incidence per 100,000 person-year of the studied intestinal parasites in Mexican children (5-9y)

As shownin Table 1, the incidence (cases per 100 000 persons/year in children from 5 to 9 years) of Age of *A. lumbricoides* was of 153.1 (SD = 211.1), the incidence of *E. histolytica/dispar* was of 549.3 (SD = 325.1) and the incidence of *G lamblia* was of 35.2 (SD-35.8). The incidence of *A. lumbricoides* and *E. histolytica* was highest in the southern states of Veracruz, Tabasco, Yucatan and Oaxaca and the incidence of *G. lamblia* was highest in the northern states of Baja California and Sinaloa and the southern state of Yucatan (Fig. 1).

**Table 1:** Incidence of the most common intestinal parasites in children (5 to 9 years) and socioeconomic and geographical characteristics of Mexican states according to their latitude

	South			North			Overall			*P*
	Mean	S.D.		Mean	S.D.		Mean	S.D.		
Incidence of *A. lumbricoides*	215.8	14.13		90.3	153.66		153.08	211.1		0.09
Incidence of *E. histolytica/dispar*	674.3	345.6		424.3	256.21		549.3	325.1		0.03
Incidence of *G. lamblia*	25.9	14.1		44.59	47.7		35.3	35.8		0.12
Mean minimum temperature (°C)	10.25	± 8.45		10.63	± 4.18		10.44	± 6.56		0.87
Mean maximum temperature (°C)	26.38	± 4.08		25.38	± 2.03		25.88	± 3.21		0.39
Average annual temperature (°C)	18.31	± 5.25		18.00	± 2.66		18.16	± 4.10		0.83
Mean minimum precipitation (mm)^a^	625	± 284		269	± 135		447	± 284		0.00
Mean maximum precipitation (mm)^a^	2681	± 1307		1519	± 999		2100	± 1287		0.01
Average annual precipitation (mm)^a^	1653	± 698		894	± 534		1273	± 723		0.00
Households living in poverty (%)	51.90	± 14.01		40.05	± 10.23		45.98	± 13.48		0.01
Households living in extreme poverty (%)	14.46	± 10.55		6.98	± 3.67		10.72	± 8.65		0.01
Households without sewage system (%)	10.08	± 8.05		8.59	± 4.96		9.33	± 6.62		0.53
Households without piped water (%)	15.15	± 10.66		7.21	± 3.86		11.18	± 8.86		0.01
Households without toilet (%)	2.12	± 1.44		2.01	± 1.12		2.06	± 1.27		0.81
Population with no health coverage (%)	57.64	± 12.21		68.90	± 4.04		63.27	± 10.62		0.00
Mean Age (years)	25.25	± 1.95		25.56	± 0.96		25.41	± 1.52		0.57
Surface with warm-humid climate (%)	53.87	36.10		20.60	28.70		37.23	36.26		0.47

^a^mm per year

The states of the north and south of Mexico had similar temperatures, but they differed in the amount of precipitation: states of the south of Mexico had a higher average annual precipitation. Also states in the south had higher rates of households living in poverty and extreme poverty, and higher rates of households without access to piped water, and lower rates of health coverage of the population than their northern counterparts (Table 1).

As seen in the univariable models in Table 2, most geographical, environmental and socioeconomic variables were associated with the incidence of *A. lumbricoides,* and *E. histolytica/dispar* (Table 2), except for mean age of the population and mean altitude of the state for *A. lumbricoides*, and the mean altitude of the state for *E. histolytica/dispar.* For *G. lamblia* infection, only the latitude and altitude of the state was associated with the incidence.

**Table 2:** Linear regression models between *A. lumbricoides* incidence rates with state-wide socioeconomic environmental and geographical variables

	*Ascaris lumbricoides*			*Entamoeba Histolytica/dispar*			*Giardia lamblia*		
	β	95% C.I.	*P*	β	95% C.I.	*P*	β	95% C.I.	*P*
Latitude (°)	− 24.73	(− 43.9 to − 5.55)	0.01	− 72.97	(− 100 to 35.9)	<0.01	3.88	(0.29 to 7.46)	0.03
Mean altitude of the state (m)	− 0.07	(− 0.15 to 0.02)	0.15	− 1.26	(− 31,00 to 0.06)	0.18	− 0.01	(− 0.3 to 0,00)	0.09
Mean minimum temperature (°C)	17.68	(7.65 to 27.7)	<0.01	33.63	(10.95 to 56.31)	< 0.01	1.15	(− 9.9 to 3.29)	0.28
Mean maximum temperature (°C)	29.27	(7.32 to 51.22)	0.01	61.45	(13.71 to 109.18)	0.01	0.62	(− 3.8 to 5.07)	0.77
Average annual temperature (°C)	31.65	(16.49 to 46.81)	0.00	61.98	(27.58 to 96.44)	< 0.01	1.67	(− 1.77 to 5.1)	0.33
Mean minimum precipitation (mm)*	0.27	(0.02 to 0.53)	0.04	0.97	(0.51 to 1.45)	< 0.01	− 0.03	(− 0.08 to 0.01)	0.16
Mean maximum precipitation (mm)*	0.06	(0,00 to 0.12)	0.04	0.16	(0.05 to 0.28)	< 0.01	0.00	(− 0.01 to 0.01)	0.79
Average annual precipitation (mm)*	0.11	(0.01 to 0.21)	0.03	0.33	(0.13 to 0.53)	< 0.01	0.00	(− 0.02 to 0.02)	0.97
Surface with warm-humid climate (%)	3.41	(2.62 to 4.25)	< 0.01	8.21	(4.66 to 11.76)	< 0.01	− 0.08	(− 0.47 to 0.32)	0.69
Households living in poverty (%)	7.33	(2.18 to 12.49)	0.01	20.14	(10.01 to 30.26)	< 0.01	− 0.52	(− 1.56 to 0.53)	0.31
Households living in extreme poverty (%)	14.53	(7.22 to 21.84)	< 0.01	34.61	(19.78 to 49.43)	< 0.01	− 0.48	(− 2.13 to 1.16)	0.55
Households without access to sewage system (%)	4.74	(8.84 to 28.19)	< 0.01	39.36	(18.3 to 60.42)	< 0.01	− 0.02	(− 2.18 to 2.14)	0.20
Households without access to piped water (%)	16.34	(9.88 to 22.81)	< 0.01	39.07	(26.59 to 51.66)	< 0.01	0.14	(− 1.48 to 1.17)	0.18
Households without access to toilet (%)	38.74	(25.75 to 51.73)	< 0.01	63.67	(29.06 to 98.2)	< 0.01	− 2.17	(− 5.6 to 1.27)	0.98
Population with no health coverage (%)	3.49	(− 13.92 to − 0.05)	0.05	− 13.95	(− 29.08 to 1.19)	0.07	0.52	(− 1.48 to 1.75)	0.95
Mean age (years)	− 34.97	(− 85.04 to 15.1)	0.16	− 26.84	(− 42.46 to − 11.32)	< 0.01	4.93	(− 10.55 to 20.24)	0.86

For *A. lumbricoides*, the final multivariable model with a goodness-of-fit of 77% (R^2^ = 0.77), showed that the mean minimum temperature of the state, the state-wide rate of households without access to piped water and the rate of households without access to a toilet are the variables that best fit the incidence of *A. lumbricoides* incidence (Table 3).

**Table 3:** Best multivariable model for each intestinal parasitic infection incidence

	β	95% C.I.	*P*
*Ascaris lumbricoides*			
Mean minimum temperature (°C)	13.24	(7.90–18.58)	0.03
Households without access to piped water (%)	12.22	(5.02–19.42)	0.02
Households without access to toilet (%)	23.96	(12.36–35.59)	< 0.01
R squared (0.77)			
*Entamoeba Histolyticadispar*			
Mean minimum precipitation (mm)	1.02	(0.61–1.43)	< 0.01
Households without access to a toilet (%)	25.33	(−2.80–53.48)	0.07
Households without access to sewage system (%)	35.69	(14.35–57.03)	< 0.01
Households without access to piped water (%)	18.28	(−0.58–37.14)	0.05
R squared (0.73)			
*Giardia lamblia*			
Latitude (°)	3.88	(0.29–7.46)	0.03
R squared (0.11)			

Multivariate analysis for *E. histolytica/dispar* revealed that mean minimum precipitation in the state, the rate of households without access to a toilet, without access to piped water and without access to sewage system best fit the incidence of *E. histolytica/dispar* infections (Table 3). The final model explained 73.7% of the variation in the outcome.

A multivariable model for *G. lamblia* infection incidence could not be constructed, since only latitude and mean altitude of the state were associated in the univariable analysis. Only latitude was included after backward selection of the variables. This model explained 11.1% of the incidence of *G. lamblia* infections.

The internal validation analysis showed that the goodness-of fit did not change more than 10% and all the included variables remained significantly associated with each of the three intestinal parasites.

## Discussion

The present study shows the distribution of the most common intestinal parasites in Mexican children aged 5–9 years. The best multivariable model for *A. lumbricoides* and *E. histolytica/dispar* included both socioeconomic and environmental factors and explained 73.7% of the incidence, while the model for *G. lamblia* only included the latitude of the state and explained 11% of the incidence.

Availability of sanitation facilities and water supply have shown to decrease the risk of intestinal protozoa and STH, as demonstrated by a large number of studies summarized in three systematic reviews and meta-analyses (Ziegelbauer et al. 2012; Strunz et al. 2014; Speich et al. 2016). Similarly, in this study, the rate of households without a toilet and households without piped water were included in the models explaining the incidence of both *A. lumbricoides* and *E. histolytica/dispar.* A possible explanation for this association is that in Mexico households without access to piped water usually obtain water from shared water pipes, rivers, springs or water trucks “pipas”. They store the water in 200 l plastic barrels or 1000 l containers called “tinacos”. These households use this water for cooking, washing hands and drinking (Rai et al. 2000), increasing the likelihood of contamination from hands to stored water in the household (Jonnalagadda and Bhat 1995; Cruz et al. 2012).

Environmental factors showed to be important predictors for the incidence of *A. lumbricoides* and *E. histolytica/dispar,* even after including the well-established socioeconomic risk factors as potential variables in the model (Ziegelbauer et al. 2012; Strunz et al. 2014; Speich et al. 2016). For *A. lumbricoides,* the multivariable model showed that higher state-wide mean minimum temperature was associated with higher *A. lumbricoides* incidence. *A. lumbricoides* eggs require temperatures between 28 and 32 °C to complete embryoogenesis (Gaasenbeek and Borgsteede 1998), lower temperatures slows *A. lumbricoides* egg development and reduces the number of eggs that become infective (Dziekonska-Rynko and Jablonowski 2004). The state-wide mean minimum annual precipitation was associated with *E. histolytica/dispar.* Indeed, rainfall provides a suitable environment for survival and mobility of *E. histolytica/dispar* (which is a waterborne parasite), increasing the likelihood of infection(Bray and Harris 1977).

The only factor associated with *G. lamblia* infection incidence in Mexican children was the latitude of the state. However, the model only explained 11% of the incidence of this parasite. In contrast to our results, other studies assessing the risk factors for *G. lamblia* at individual level have shown that low education, lack of sewage system and toilets are associated with this parasite as well (Cifuentes et al. 2000). These differences might be attributable to the design of the study, ecological modelling may not be the best approach to study *G. lamblia*. *G. lamblia* incidence is influenced by unpredictable outbreaks, usually associated with contaminated food and water sources, these outbreaks could potentially change the geographical distribution of the parasite despite the socioeconomic and environmental risk factors (Wearing et al. 2005).

This study has limitations that need to be addressed for proper interpretation of the results. There are other factors that are known to affect the incidence of intestinal parasites and were not measured, such as malnutrition and migration (Buonsenso et al. 2019). SINAVE data is based on diagnostic results of children for whom the parents were seeking health care, the real incidence of the studied parasites is most likely underestimated. Also, this study provides group-level information on the outcome and the determinants, without knowing whether these associations hold true at an individual level (i.e. ecological fallacy). In addition, it was not possible to adjust by sex of the children since the SINAVE database only provides information by age group. Therefore the results should be interpreted at state-level (Campbell et al. 2017). The major strength of the current study is that INEGI data is representative of the Mexican population at national and state level. In addition the parasite infection data of the SINAVE is collected following the same procedures nationwide and is therefore a good measuring tool for comparison purposes.

## Conclusion

In addition to the well-known socioeconomic factors contributing to the incidence of *A. lumbricoides* and *E. histolytica/dispar* we found that temperature and precipitation were associated with higher risk of infection. More research is needed to evaluate the effect of climate change on the incidence of *A. lumbricoides* and *E. histolytica/dispar*.

## Data Availability

The data used in this manuscript is publicly available.
